# Diagnostic Accuracy of Different Computed Tomography Signs for Differentiating Between Malignant and Cirrhotic Ascites Keeping Ascitic Fluid Cytology as Gold Standard

**DOI:** 10.7759/cureus.20254

**Published:** 2021-12-07

**Authors:** Ibtesam Zafar, Ayesha Isani Majeed, Muhammad Waseem Asad, Amir Khan, Muzammil Rasheed Bhutta, Muhammad Nasir Naeem Khan

**Affiliations:** 1 Radiology, Pakistan Institute of Medical Sciences, Islamabad, PAK; 2 Gastroenterology and Hepatology, Pakistan Institute of Medical Sciences, Islamabad, PAK

**Keywords:** ct scan, malignant ascites, ascitic fluid density, cirrhosis, peritoneal carcinomatosis

## Abstract

Objective

The goal of this research was to define the diagnostic precision of CT signs to distinguish malignant ascites from cirrhotic ascites. Ascitic fluid cytology was kept as the gold standard.

Study design

This research was a prospective cross-sectional study.

Place and duration of the study

Participants’ recruitment started on July 15, 2021, and the whole study lasted about three months till October 15, 2021, at the Radiology Department of Pakistan Institute of Medical Sciences, Islamabad.

Patients and methods

A total of 80 patients were included in the research and divided into two groups grounded on the cirrhotic or malignant etiology of the ascites based on their fluid cytology. Ascites volume, relative spread between the lesser sac and greater peritoneal cavity, the wall thickness of gallbladder, density of ascites, parietal peritoneum thickness and degree of its enhancement, and presence of septa and loculations were some of the major CT signs studied.

Results

The average age of patients included in this study was 36.2 ± 6.67 years (range 29-49 years). Of the 80 patients, 50 (62.5 %) were men, and 30 (37.5 %) were women. CT signs associated with the malignant ascites reported in this study were fluid present in the lesser sac (p = 0.03), peritoneal thickening and degree of its enhancement (p = 0.05), increased ascites density (p= 0.001), and presence of septa and loculations (63.6 % of malignant ascites). However, gallbladder wall thickness did not show any variation between both groups.

Conclusion

We conclude that in the diagnosis of malignant ascites, CT scan imaging can play a vital role. This research approves and testifies the benefits of indirect signs such as the spread of ascites, increased density of ascites, thickening and enhancement of parietal peritoneum, and ascitic fluid complexity in pointing out malignancy as a cause of ascites.

## Introduction

Ascites is a frequent finding in a number of pathologic disorders including hepatic failure, cardiac dysfunction, metabolic imbalances, inflammatory processes, and neoplasms leading to accumulation of free fluid in the peritoneal cavity [1]. Hepatic cirrhosis is undoubtedly the most frequent etiology of ascites, single-handedly accountable for over and above three-quarters of cases [2]. The main differential is the ascites due to malignant etiology accounting for about 10% of cases and the remaining 5% of cases are attributed to other causes [1,2]. The recognition of ascites as an oncologic challenge is always problematic, the difficulty being to distinguish between the cause of ascites as hepatic cirrhosis or malignancy.

Ascites, the atypical buildup of fluid in the peritoneal cavity, develops in approximately 60% of individuals having hepatic cirrhosis within a span of about 10 years after diagnosis. It suggests a poor prognosis associated with a high mortality rate ranging from 40% within a year to 50% within two years. In the case of intractable ascites, the median duration of survival does not surpass six months, which is owing to the development of serious pathologies. The factors associated with the worst prognosis are hyponatremia and bacterial peritonitis, low total protein absorption, and raised number of red cells in ascitic fluid analysis over and above 10,000/mm^3^ (hemorrhagic ascites) [2,3].

Ascites in hepatic cirrhosis is produced by multiple physiological and pathological disturbances characteristic of portal hypertension [4]. Reduction in glomerular filtration rate and kidney dysfunction with sodium and water retention is caused by splanchnic and systemic arterial vasodilation together with the stimulation of various neural and hormonal mechanisms. In addition, the deranged general hemodynamic conditions bring about the development of ascites, dilutional hyponatremia, and hepatorenal syndrome progression. In many pathologic disorders, ascites represents a late expression and/or a complication of the illness, while in others, it signifies one of the earliest indicators of the pathophysiology intricated. Ascites might be the primary reason for the patient’s having sought medical aid and its discovery and assessment is an important factor in diagnosis. Computed tomography (CT) images can portray a little amount of ascites, particularly in the initial stages when the fluid gathers in the peritoneal reflections or perihepatic and perisplenic sites.

The role of the radiologist is being able to illustrate small pockets of fluid while they are confined in specific perivisceral spaces sorting out the etiologic procedure into intra- vs. extraperitoneal sites [5,6], and distinguishing massive ascites due to benign causes, neoplastic process, or a grouping of both [7,8]. In this fashion, the fundamental pathology might be diagnosed earlier or complications of underlying disease might be cured.

The usual workup when ascites is encountered in the clinical setting is a grouping of clinical history, physical examination, blood and urine assessments, sonography of abdomen, and abdominal paracentesis. CT scan is not considered generally as a primary investigation in case of ascites unless combined with other factors; however, ascites is frequently encountered as an incidental finding in many patients undergoing CT imaging. In its present condition, imaging single-handedly is not in a spot to be a reliable tool for distinguishing between benign and malignant etiology of ascites; however, it is proposed that multiple CT signs may prove helpful in picking out the cause of ascites [9-12].

The purpose of this research was to define the diagnostic precision of the different CT signs in the determination of the cause of ascites being cirrhosis or malignancy whereas ascites fluid cytology is kept as the gold standard.

## Materials and methods

This study was approved by the Ethical Review Board of Shaheed Zulfiqar Ali Bhutto Medical University/Pakistan Institute of Medical Sciences, Islamabad (F.1-1/2015/ERB/SZABMU/809). Informed written consent as sanctioned by the research department was attained from all the participants of the study. Participants were recruited from the Radiology Department of Pakistan Institute of Medical Sciences, Islamabad. Contributors’ enrollment started on July 15, 2021, and the entire study lasted about three months till October 15, 2021.

A total of 80 patients who were referred to the radiology department for contrast-enhanced CT scan of the abdomen, having clinical suspicion of ascites, were enrolled in the study. Patients aged 25 years old or above were included in the study. While exclusion criteria were patients with known causes of ascites other than cirrhosis or malignancy like cardiac or renal disease, patients coming for follow-up scans, and patients with known contrast allergy. Pediatric patients with ascites were also excluded from the study.

Subjects were allocated to a group centered on ascites etiology resulting in the formation of two groups. Group 1 included patients with cirrhosis established by liver imaging, histopathology, and abdominal paracentesis, and Group 2 included patients with peritoneal carcinomatosis established by abdominal paracentesis followed by cytology of ascitic fluid executed in advance or recommended in the course of the CT scan. Many forms of tumors are attributed to the cause of ascites including pancreatic, ovarian, colon, gastric, renal, breast, and some others.

The exam was performed with a multislice OPTIMA CT scanner (GE Healthcare, Chicago, IL). The consistent protocol encompassed an abdominopelvic bulk acquisition of 90 seconds afterward IV injection of 100 milliliters of iodinated contrast iohexol (Omnipaque^(R)^ injection 350 mgI/ml) with a Nemoto jet injector at a stream rate of 3 ml/s. A dual-phase CT scan was performed. The porto-venous phase was taken at 60-70 seconds and the delayed phase was taken at a five-minute interval. The scans were reported by a radiologist based on CT scan signs shown in Table 1.

**Table 1:** Different CT signs included in the study

CT signs
Density of the ascites
Ascites dimensions and comparative scattering between the lesser sac and greater peritoneal cavity
Enhancement and thickness of peritoneum
Gallbladder wall thickness
Presence of septa and loculations

The density of ascites was calculated by placing the region of interest (ROI) in the center of the ascitic mass at least 3 cm from the boundary. Three measurements were taken and mean density was calculated.

The clinical result of every patient was established on the basis of ascitic fluid cytology. The prevalence of signs mentioned in Table 1 was then studied in these patients and a comparison was made between two groups. Data were recorded and statistical analysis was performed using the Statistical Package for Social Sciences, version 21 (IBM SPSS Statistics, Armonk, NY). A p-value <0.05 was considered significant.

## Results

A total of 80 patients were included in the study. The mean age of the patients enrolled in the study was 35.8 ± 7.74 years (ranging from 25 to 49 years). Out of the 80 patients, 50 (62.5%) patients were men, and 30 (37.5%) were women (Table 2).

**Table 2:** Baseline demographic characteristics of study participants.

Variables	Subjects (n = 80)
Age	35.8 ± 7.74
Gender	
Male	50 (62.5%)
Female	30 (37.5%)

We divided the subjects into two groups on the basis of ascites etiology. Group 1 consisted of cirrhosis patients established by liver histopathology and fluid cytology while Group 2 consisted of peritoneal carcinomatosis patients established on the basis of fluid cytology. Group 1 (cirrhotic ascites) comprised a total of 36 individuals out of which 24 were men and 12 were women. Group 2 (malignant ascites) comprised a total of 44 individuals out of which 26 were men and 18 were women.

A total of 22 patients (61%) showed no fluid in the lesser sac and 14 (39%) patients showed fluid in the lesser sac in Group 1. No fluid was present in the lesser sac whenever the quantity of ascites was little or moderate in the peritoneal cavity (16 of 36 cases, i.e., 44% cases) of cirrhotic ascites. Fluid was seen in the lesser sac only when there was a large amount of fluid in the peritoneal cavity (56%). The lesser sac was still empty in six of 20 such patients (30%). In Group 2, fluid was seen in the lesser sac in 38 out of 44 patients; however, no fluid was seen in the lesser sac in six out of 44 participants, i.e., 7.5%. Fluid in the peritoneal cavity was in a lesser amount in 17 out of 44 patients (38.6%) and in a greater amount in 21 out of 44 patients (47.7%). These statistics show that the occurrence of liquid in the lesser sac was associated with malignancy as a cause of ascites and is not just related to the total amount of fluid in the peritoneal cavity (p = 0.004) (Table 3).

**Table 3:** Characteristics of ascites in both groups and diagnostic performance of CT scan.

Characteristic features	Cirrhotic (Group 1; n = 36)	Malignant (Group 2; n = 44)
Gender		
Male	24	26
Female	12	18
Fluid in the lesser sac		
Empty	22	6
Fluid-filled	14	38
Density of ascites	5.69 ± 3.05 HU	11.35 ± 3.36 HU
Gallbladder wall thickness	3.5 mm	3.1 mm
Presence of septa and loculations	47. 2%	63.6%

**Figure 1:**
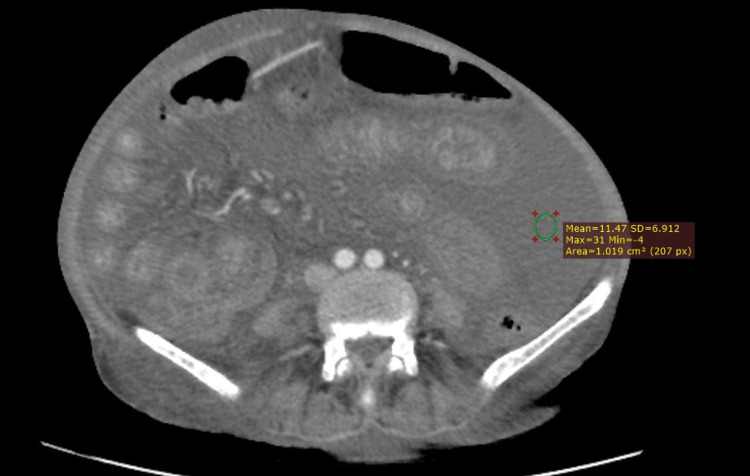
Measurement of ascitic density in a patient with known ovarian malignancy.

The mean density of ascites was lesser in patients with cirrhosis (mean attenuation 5.69 ± 3.05 HU) than the patients with malignancy (mean density 11.35 ± 3.36 HU) as a cause of ascites as shown in Figure 1. A significant difference could be seen in both groups (p = 0.001).

**Figure 2:**
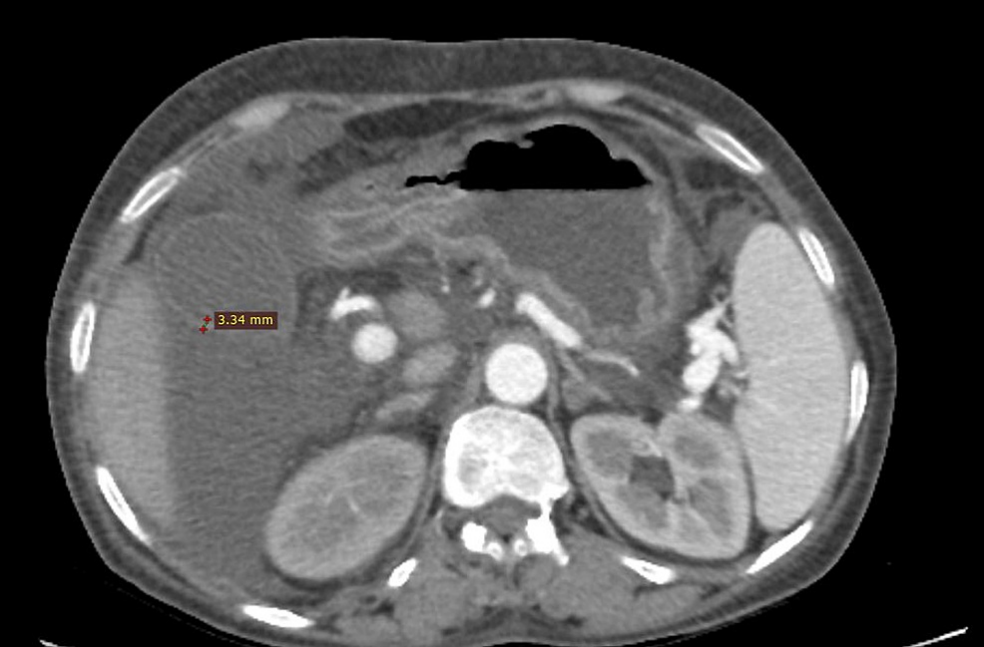
Measurement of gallbladder wall thickness in a patient with cirrhotic ascites.

The mean wall thickness of the gallbladder as shown in Figure 2 was 3.5 mm in Group 1, while in Group 2, it was 3.1 mm. No significant difference was observed between these two groups (p = 0.42). Gallbladder wall thickness could not be evaluated in patients with a history of cholecystectomy (n = 3) or patients already having gallbladder pathologies (n = 1).

In malignant ascites, thickening of parietal peritoneum with delayed enhancement was recorded more frequently (p = 0.004) as compared with cirrhotic ascites.

The presence of septa and loculations was considered as a marker or complexity of ascites. In Group 1, septa and loculations were seen in 17 out of 36 patients, while in Group 2, they were recorded in 28 out of 44 patients. Hence, the complexity of ascites was more frequently seen in malignant ascites (63.6%) than the cirrhotic ascites (47.2%).

## Discussion

Ascites denotes the presence of free fluid in the peritoneal cavity. Numerous studies have concluded that the presence of ascites in the lesser sac is not a characteristic manifestation of widespread ascites and that it must lead to analysis for the participation of adjacent organs or peritoneum for neoplastic development [11-13]. Our study confirms these conclusions by observing different ascites distribution grounded on etiology. In our study, we found that fluid was present in the lesser sac in the majority of the patients with malignant ascites even when a little amount of fluid was seen in the peritoneal cavity. In contrast, in cirrhotic ascites associated with portal hypertension, fluid was rarely found in the lesser sac except for the cases where there was a very large amount of ascites in the peritoneal cavity. We agree with the results as stated by Gore et al. [11] that there is not complete freedom for fluid transmission between these two areas, despite their theoretical association through the epiploic foramen.

The density of ascites is usually considered as a parameter for assessing the composition of ascites. Exudates usually show higher attenuation on CT scans as they have a higher protein content as compared to transudates, which show lower attenuation. Topal et al. [12] stated in their study that the density of ascites can be used as a reliable tool in differentiating between exudative and transudative ascites with a sensitivity of 68%. We found a significant difference between the density of cirrhotic and malignant ascites in our study stating a considerable higher density of malignant ascites, which again proves this sign to be beneficial in the determination of the etiology of ascites.

Previous studies have shown that the gallbladder wall thickness might prove to be a useful parameter in pointing out the malignant etiology of ascites. Tsujimoto et al. [14] stated that 95% of the cases with normal or thin-walled gallbladders were shown to have malignant ascites while 82% of cases with thick-walled gallbladders were discovered to have cirrhosis as a cause of ascites. The results of our study, which find no significant differences in gallbladder wall width between both groups, do not coincide with these findings. The possible cause of this discrepancy might be based on the fact that previously ultrasound was employed in the measurement of gallbladder wall thickness while we used CT scan for this purpose [14,15].

Peritoneal enhancement with increased thickness is the second most common sign associated with the neoplastic process involving the peritoneum, the most common being the presence of ascites [16-18]. Risson et al. [9] stated in their study that peritoneal thickening with enhancement is more commonly associated with malignant ascites. Our study also shows a significant p-value of 0.004 associated with peritoneal thickening and enhancement, which is in accordance with previous literature available.

In our research, we found a higher incidence of the presence of septa and loculations in patients with malignant ascites as compared with patients having cirrhotic ascites. This appears to be in accordance with the fact that malignant ascites comprised exudates containing more proteins and cellular material as compared with transudative ascites, which forms the base of fluid in patients with cirrhosis. The presence of more protein and cellular material adds to the complexity of the ascites, which is radiologically visible as septa and loculations. This testifies the benefit of this sign in the evaluation of the cause of ascites. In previous studies, this sign was mostly described on ultrasound; however, in our study, a CT scan was employed to evaluate the complexity of ascites.

Some of the limitations in our study include a single spectator with a small cohort and short duration, which excludes any reproducibility in the study. Another limitation of our study includes that we used a single modality and no comparison was made between different modalities. Stefan et al. [19] and Zhang et al. [20] proposed the use of new modalities like magnetic resonance imaging and positron emission tomography-computed tomography as useful new methods in differentiating benign vs. malignant etiology of ascites. At present, these modalities are not cost-effective and are not commonly available so they are usually reserved only for complex cases. In the future, they might prove to be useful as a routine workup for ascites.

## Conclusions

Diagnostic workup for ascites does not usually include CT scan as a part of investigations; however, it has a significant contribution in discriminating the cause of ascites when performed. Our study confirms the utility of indirect CT signs like the spread of ascites, increased density of ascites, thickening and enhancement of parietal peritoneum, and ascitic fluid complexity in pointing out malignancy as a cause of ascites. Results of this study suggest that description of these signs should be employed by radiologists in routine reporting when ascites is encountered. This might prove beneficial for clinicians in the initiation of necessary workup in case of malignant ascites and would result in the reduction of unnecessary investigations in case of benign etiology of ascites.
